# Anti-infection roles of miR-155-5p packaged in exosomes secreted by dendritic cells infected with *Toxoplasma gondii*

**DOI:** 10.1186/s13071-021-05003-x

**Published:** 2022-01-06

**Authors:** Dan Jiang, Shuizhen Wu, Liqing Xu, Guantai Xie, Dongliang Li, Hongjuan Peng

**Affiliations:** Department of Pathogen Biology, Guangdong Provincial Key Laboratory of Tropical Disease Research, School of Public Health, Southern Medical University, Guangzhou, 510515 Guangdong Province China

**Keywords:** *Toxoplasma gondii*, Exosomes, RAW264.7 cells, miR-155-5p, SOCS1

## Abstract

**Background:**

*Toxoplasma gondii* is a zoonotic intracellular protozoon that is estimated to infect about 30% of the world’s population, resulting in toxoplasmosis in immunocompromised patients and adverse outcomes in cases of primary infection during pregnancy. Exosomes are tubular vesicles secreted by cells, and function in intercellular communication. It has been reported that the exosomes secreted by *T. gondii-*infected immune cells transmit infection signals to the uninfected cells. However, the mechanism and effect of the exosome transmission are still vague. We therefore investigated the function of the exosomes transmitted from DC2.4 cells infected with the *T. gondii* RH strain (*Tg*-DC-Exo) to the uninfected cells, as well as their roles in anti-infection.

**Methods:**

We conducted exosome isolation and identification with ultracentrifugation, transmission electron microscopy (TEM), nanoparticle tracking analysis (NTA), and western blot (WB) analysis. Exosome uptake by recipient cells was identified by PKH67 assay. The signal transmission and the abundance of miR-155-5p were determined using transwell assay and qRT-PCR. For detection of immune responses, cytokine secretion was evaluated. The *T. gondii* B1 gene was determined to evaluate tachyzoite proliferation.

**Results:**

We observed that *Toxoplasma* infection upregulated miR-155-5p expression in DC2.4 cell-secreted exosomes, and those exosomes could be ingested by murine macrophage RAW264.7 cells. *Tg*-DC-Exo and miR-155-5p stimulated host proinflammatory immune responses including increased production of proinflammatory cytokines IL-6 and TNF-α, and proinflammatory marker-inducible nitric oxide synthase (iNOS). The NF-κB pathway was activated by downregulation of SOCS1, leading to inhibition of *T. gondii* tachyzoite proliferation in RAW264.7 cells.

**Conclusions:**

Our findings provide a novel mechanism for how infected cells transmit infection signals to the uninfected cells through exosome secretion after *T. gondii* infection, followed by inflammatory responses and anti-infection reactions, which may help us develop a new strategy for toxoplasmosis prevention, especially in immunocompromised patients.

**Graphical Abstract:**

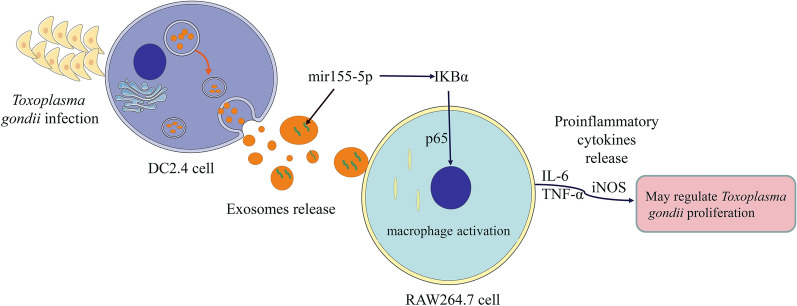

**Supplementary Information:**

The online version contains supplementary material available at 10.1186/s13071-021-05003-x.

## Background

*Toxoplasma gondii* is an obligate intracellular protozoon that infects almost one third of the world’s population [1]. The infection is usually asymptomatic, but poses a lifelong threat to immunocompetent populations; however, it may result in severe toxoplasmosis and even death in immunocompromised patients, and primary infection during pregnancy may result in adverse pregnancy outcomes [1]. Host immune responses mediate the effects of *T. gondii* infection [2]. For example, interferon-gamma (IFN-γ), interleukin-12 (IL-12), and IL-1α have been reported to promote protective immunity against *T. gondii* infection [3–5]. Macrophages are important components of the innate immune system; they scavenge pathogens by activating innate immunity or promoting adaptive immunity through antigen presentation [6]. Many *T. gondii* secretions, including ROP16, GRA12, GRA15, and GRA2, have been reported to regulate the host immune response [7–9].

Exosomes are 30–200 nm extracellular vesicles enriched in proteins, nucleic acids, glycoconjugates, lipids, and other biological substances, which are powerful tools for intercellular communication that mediate biological, physiological, and pathological states related to immune responses [10, 11]. Most cells and pathogens can secrete exosomes containing unique surface molecules and contents including proteins, microRNAs (miRNAs), and messenger RNAs (mRNAs), which participate in host pathogenesis [12, 13]. It has been reported that *T. gondii* exosomes can trigger humoral and cellular immune responses and promote the secretion of proinflammatory cytokines by macrophages [14, 15]. *Toxoplasma gondii* exosome immunization was found to be capable of inducing immune protection to prolong mice survival [16, 17]. Nematode exosomes can inhibit the innate type 2 response [18]. *Schistosoma mansoni* exosomes contain schistosome-derived miRNAs and proteins involved in host–parasite interactions [19]. It has been shown that exosomes have potential for use in vaccine development and therapeutic innovation [20, 21].

Small non-coding RNAs, or microRNAs (miRNAs), inhibit the transcription and translation of the target genes, mainly by binding to the 3′-UTR regions. Exosomes released by immune cells can carry miRNAs from donor cells to recipient cells to regulate biological processes [22]. Rather than regulating their own cells, exosomal miRNAs can regulate proximal and distal target cells through exosome transportation or other means. However, how exosomal miRNA works has not been fully characterized. Several highly expressed miRNAs have been identified from the extracellular vesicles of *Schistosoma japonicum* and have been found to be able to suppress target gene expression in RAW264.7 cells [23].

The activation of CD8^+^ T cells is essential for inhibition of *T. gondii* infection [24]. Dendritic cells and macrophages are important antigen-presenting cells for T cells, which play important roles in anti-infection [25, 26]. The communication between these immune cells is particularly important. However, the exact mechanism by which the exosomes secreted by dendritic cells transmit the anti-infection signals to macrophages is not totally established. This study was conducted to answer this question.

## Methods

### Parasites and cell lines

Murine dendritic cell line DC2.4, human foreskin fibroblast (HFF) cell line, and murine macrophage cell line RAW264.7 were purchased from the American Type Culture Collection (ATCC, Manassas, VA, USA) and preserved in our laboratory. The DC2.4 cells were cultured in RPMI-1640 (Roswell Park Memorial Institute-1640; Gibco/Invitrogen, Waltham, MA, USA); HFF and RAW264.7 cells were cultured in Dulbecco’s modified Eagle’s medium (DMEM) (Gibco/Invitrogen). Both of the culture mediums were supplemented with 10% fetal bovine serum (FBS) (Gibco/Invitrogen) and 1% gentamicin (10 mg/ml, Invitrogen, USA). The cells were cultured with 5% CO_2_ at 37 °C. The *Toxoplasma gondii* RH strain was propagated in HFF cells in our laboratory.

### Exosome isolation

DC2.4 cells were cultured with RPMI-1640 complete medium supplemented with 10% exosome-free FBS (Gibco) in eight 75 cm^2^ cell culture flasks to 90–100% confluence; four flasks were infected with RH tachyzoites at a multiplicity of infection (MOI) of 3 for 0.5 h, and the unrecruited tachyzoites were washed off three times with PBS. After that, the eight flasks were cultured with the exosome-free RPMI-1640 complete medium for 28 h. The supernatants were centrifuged at 2000×*g* at 4 °C for 30 min to remove cells and cell debris, followed by centrifugation at 10,000×*g* at 4 °C for 45 min to remove insoluble particles, and then ultracentrifugation at 100,000×*g* at 4 °C for 120 min to harvest the exosomes. Ultracentrifugation was repeated once, and the supernatant was carefully aspirated. The precipitates were resuspended in 200 µl PBS and stored at −80 °C for use [27].

### Exosome identification

Isolated exosomes were examined using transmission electron microscopy (TEM) (Hitachi, Tokyo, Japan) at a voltage of 80 kV. Exosomal proteins were detected with western blotting (WB) [27], and the exosome size was determined using a ZetaView^®^ nanoparticle tracker (Particle Metrix, Germany) as described previously [28]. The ZetaView combines classic microelectrophoresis techniques with Brownian motion to provide information about particle size, zeta potential, and particle concentration. Briefly, the ZetaView sample cubicle was washed with distilled water and slowly injected with exosome diluent samples. The size of the exosomes was measured. Exosomal total protein concentration was determined using a Pierce BCA Protein Assay Kit (Thermo Fisher Scientific, MA, USA) according to the manufacturer’s instructions.

### Western blot

The isolated exosomes and the transfected cells were collected and lysed using lysis buffer (Beyotime Biotechnology, Shanghai, China), total proteins were loaded for sodium dodecyl sulphate–polyacrylamide gel electrophoresis (SDS-PAGE), and WB was performed as described previously [29]. The following primary antibodies were used: CD9 rabbit monoclonal antibody (mAb; 1:1000), TSG101 rabbit mAb (1:1000), HSP70 rabbit mAb (1:1000), iNOS rabbit mAb (1:1000), p65 rabbit mAb (1:1000), and SOCS1 rabbit mAb (1:1000) purchased from Abcam (MA, USA); GAPDH mouse mAb (1:1000), phosphorylated IκB alpha (Ser32/Ser36) mouse mAb (1:1000), and phosphorylated NF-κB p65 (S536) mouse mAb (1:1000) purchased from Affinity Biosciences (OH, USA); and IKBα rabbit mAb (1:1000) purchased from GeneTex (Santa Cruz, CA, USA). The secondary antibodies used for WB were horseradish peroxidase (HRP)-conjugated goat anti-mouse immunoglobulin G (IgG; 1:5000) and HRP goat anti-rabbit IgG (1:5000) purchased from ABclonal (Wuhan, China).

### Detection of uptake of *Tg-*DC-exo and DC-exo by RAW264.7 cells

RAW264.7 cells were seeded on coverslips in a 12-well plate, at approximately 1.1 × 10^6^ cells per well, and were cultured with DMEM supplemented with 10% exosome-free FBS for 12 h. Exosomes extracted from the supernatants of *T. gondii*-infected DC2.4 cells (*Tg*-DC-Exo) and DC2.4 cells (DC-Exo) were labeled with green fluorescence using a PKH67 Green Fluorescent Cell Linker Mini Kit (Sigma-Aldrich, USA) according to the experimental procedures described by Lin et al. [30]. Briefly, *Tg*-DC-Exo and DC-Exo (5 µg for each) were respectively mixed in 40 µl of PBS with 50 µl Diluent C and 0.25 µl PKH67 dye, mixed gently for 5 min at room temperature, and 1% bovine serum albumin (BSA) was added to terminate the dying process. PKH67-stained *Tg*-DC-Exo and DC-Exo were recollected at 100,000×*g* for 2 h at 4 °C and resuspended in PBS. PKH67-stained exosomes or the same volume of the PKH67-PBS control were separately added to the RAW264.7 cells and incubated for 6 h. The cells were washed three times with PBS times and fixed in 4% paraformaldehyde for 10 min (Dingguo, China). The coverslips were taken out, rinsed with double-distilled water, and air-dried. The coverslips were mounted with DAPI Fluoromount^®^ (Southern Biotech, USA) and then observed under a fluorescence microscope (Nikon, Tokyo, Japan) with a green fluorescence protein (GFP) filter, and images were captured at ×1000 magnification.

### RNA and DNA isolation and Quantitative Reverse Transcription-Polymerase Chain Reaction (qRT-PCR)

The total RNA of exosomes was extracted with an exoRNeasy Serum/Plasma Midi Kit (QIAGEN, Duesseldorf, Germany) according to the manufacturer’s protocol. The total RNA of cells was extracted with TRIzol reagent (Thermo Fisher Scientific, Waltham, MA, USA) according to the manufacturer’s protocol. Genomic DNA was removed with a One-Step gDNA Removal Kit (Trans Gen Biotech, Beijing, China). For miRNA analysis, exosomal RNA was reverse-transcribed using SuperScript™ II reverse transcriptase (Thermo Fisher Scientific, Wilmington, DE, USA), and cell RNA was reverse-transcribed using EasyScript^®^ All-in-One First-Strand cDNA Synthesis SuperMix. To evaluate the relative amount of *T. gondii* tachyzoites, the total DNA of infected cells was extracted with a DNeasy Blood and Tissue Kit, and Proteinase K (QIAGEN, Duesseldorf, Germany) was used according to the manufacturer’s protocol. Detection of the *T. gondii* B1 gene was carried out following previously reported procedures [31]. Real-time polymerase chain reaction (PCR) was performed using the Hieff^®^ qPCR SYBR^®^ Green Master Mix (Yeasen, Shanghai, China) and the QuantStudio™ real-time PCR system (Thermo Fisher Scientific, Wilmington, DE, USA). The primers for quantitative PCR (qPCR) are shown in Additional file 1: Table S1. The relative mRNA level was measured using the 2^−ΔΔCt^ method.

### Detection of macrophage polarization after treatment of RAW264.7 cells with *Tg*-DC-exo and DC-exo

RAW264.7 cells were cultured with DMEM supplemented with 10% exosome-free FBS in 24-well plates to 60–80% confluence, and *Tg*-DC-Exo or DC-Exo were added to a final concentration of 120 µg/ml. The cells were cultured for 24 h for total RNA isolation, and 48 h for total protein extraction. The total RNA isolation, complementary (cDNA) preparation, and qPCR were performed as described above. With the total protein, the proinflammatory marker (iNOS) was detected with WB.

### Transwell experiment

DC2.4 cells were seeded in the upper chambers of 24-well transwell inserts (Corning, NY, USA) and cultured with DMEM supplemented with 10% exosome-free FBS and 1% gentamicin to 100% confluence. The cells were divided into four groups: two groups were left uninfected, and two groups were infected with the *T. gondii* RH strain at MOI of 3. After infection for 30 min, the culture medium was aspirated, and the DC2.4 cells in the upper chambers were washed three times with PBS to remove the unrecruited *T. gondii* tachyzoites. Meanwhile, RAW264.7 cells were seeded in the lower chambers. The four groups of DC2.4 cells (two infected with *T. gondii* and two uninfected) in the upper chambers and the RAW264.7 cells in the lower chambers were co-cultured in DMEM supplemented with 10% exosome-free FBS and 1% gentamicin, with or without 100 µl of 10 µm GW4869 (inhibitor of exosome secretion, MedChemExpress, USA) for 52 h. These groups were labeled as normal, DC2.4 + GW4869, DC2.4 + RH, and DC2.4 + RH + GW4869. Next, the RAW264.7 cells were harvested and lysed with lysis buffer (Beyotime Biotechnology, China). The total RNAs were extracted for detection of miR-155-5p abundance via quantitative real-time PCR (qRT-PCR). Each group was prepared in triplicate, and the experiment was carried out three times for statistical analysis.

### Cell transfection with synthesized miRNA or siRNA and treatment with exosomes

RAW264.7 cells were seeded in 6-well plates to 60–80% confluence and divided into four groups. Two groups were transfected with 10 µl of 20 nm miR-155-5p mimics and mimic-normal control (miRNA mimic-NC), and miR-155-5p inhibitors and miRNA inhibitor-normal control (miRNA inhibitor-NC) (Gene Pharma, Suzhou, China), respectively. The other two groups were transfected with 10 µl of 20 nm si-*socs1* (siRNA targeting *socs1*) and si-NC (siRNA-NC) (RiboBio, Guangzhou, China), respectively. The transfection was performed using Lipofectamine 3000 (Thermo Fisher Scientific) according to the protocol provided by the manufacturer. The exosomes extracted from the cell culture supernatant of the DC2.4 (DC-Exo) and the DC2.4 infected with *T. gondii* (*Tg*-DC-Exo) were added to the 60–80% confluent RAW264.7 cells at 120 µg/well, respectively.

### Cell activity, proliferation, and polarization detection

The RAW264.7 cells were incubated with exosomes or transfected with miRNA/siRNA. At 24 h post-treatment, the transcription of the related cytokines was detected via qRT-PCR and WB, and a CCK8 kit (Cell Counting Kit-8, TransGen Biotech, Beijing, China) was used to detect the cell viability and proliferation according to the instructions. Similarly, cell proliferation activity was evaluated by detecting cell absorbance after treatment for 0, 24, 48, 72, and 96 h. Each group was prepared in triplicate, and the experiment was carried out three times for statistical analysis.

### Statistical analysis

The differences between two or three groups were analyzed with Prism (GraphPad Software) using the Student’s *t*-test and one-way analysis of variance (ANOVA). SPSS software (version 20) was used to analyze multiple comparisons (Tukey’s test), and *P* < 0.05 indicated that the difference was statistically significant.

## Results

### Characterization of the exosomes derived from DC2.4 Cells infected or uninfected with *T. gondii* RH strain

**Fig. 1:**
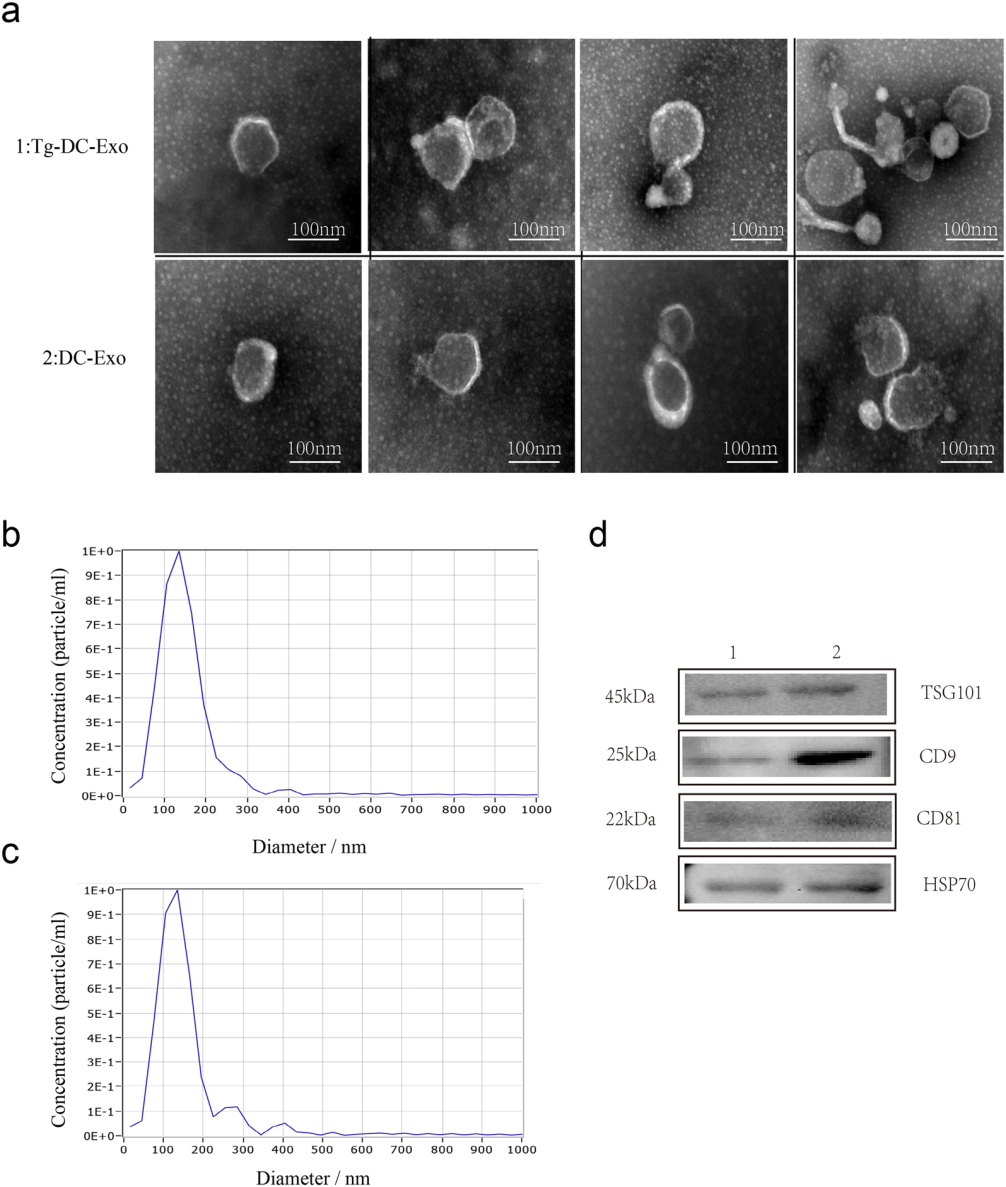
Identification of the extracted exosomes. Exosomes were extracted from dendritic cells infected with *T. gondii* (RH strain) for 28 h or uninfected cells. **a** Transmission electron microscopy was used to analyze the morphological structure of the exosomes obtained from the infected (*Tg*-DC-Exo) or uninfected dendritic cells (DC-Exo). Particle size and concentration of *Tg*-DC-Exo (**b**) and DC-Exo (**c**) were detected with nanoparticle tracking analysis (NTA). **d** Exosome-specific proteins TSG101, CD9, and CD81 and heat-shock protein HSP70 were detected in DC-Exo (1) or *Tg*-DC-Exo (2) using western blot

The exosomes extracted from the DC2.4 cells infected or uninfected with *T. gondii* presented a specific saucer structure when observed by transmission electron microscopy (TEM) (Fig. 1a). The nanoparticle tracking analysis showed that the particles had a diameter of 30–200 nm, and also revealed particle concentrations of *Tg*-DC-Exo (5.9 × 10^7^ particles/ml) and DC-Exo (3.3 × 10^7^ particles/ml) (Fig. 1b, c). Several specific exosome marker proteins, including TSG101, CD9, CD81, and HSP70, were detected in the same volume of exosome extracts with WB (Fig. 1d).

### Macrophage (RAW264.7) uptake of exosomes secreted by DC2.4 cells

**Fig. 2:**
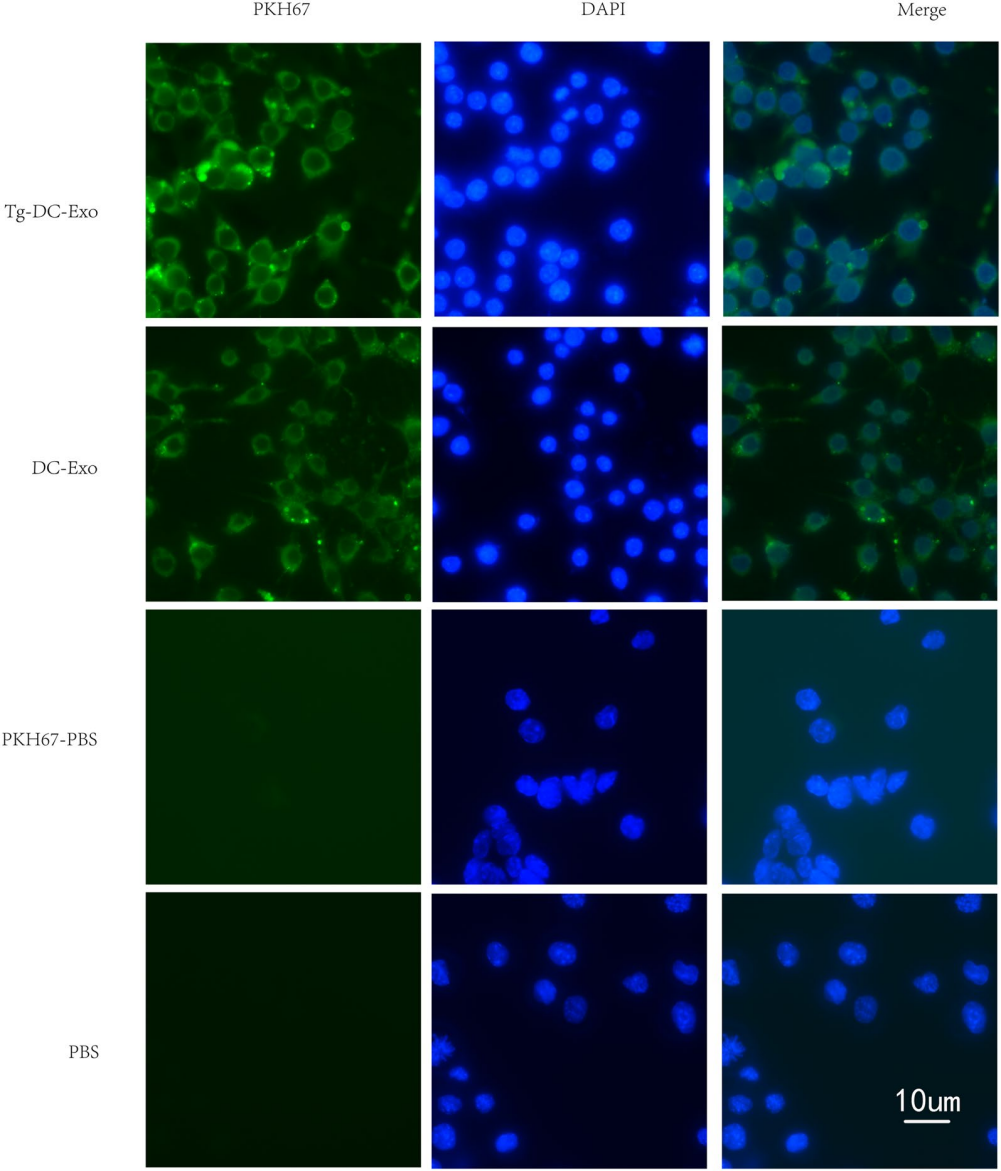
Detecting the uptake of *Tg-*DC-Exo and DC-Exo by RAW264.7 cells. *Tg-*DC-Exo, DC-Exo, and PBS were labeled using the PKH67 Green Fluorescent Cell Linker Mini Kit and added to RAW264.7 cells for 6 h incubation; PKH67-PBS and PBS were set as negative control. Green fluorescence indicates the labeled *Tg-*DC-Exo and DC-Exo. The nuclei were stained blue by DAPI. The pictures were taken at ×1000 magnification under a fluorescence microscope (Nikon, Japan)

To determine whether immune cells can communicate with each other through exosome transmission, we labeled exosomes with PKH67 and incubated them with RAW264.7 for 6 h in vitro. As the confocal microscope results show (Fig. 2), the RAW264.7 cells successfully engulfed the exosomes (including *Tg*-DC-Exo and DC-Exo) extracted from DC2.4 cells; however, this uptake phenomenon was not observed in the PKH67-PBS and PBS groups.

### *Tg*-DC-exo results in polarization of macrophages toward M1 and promotion of inflammation progress

**Fig. 3:**
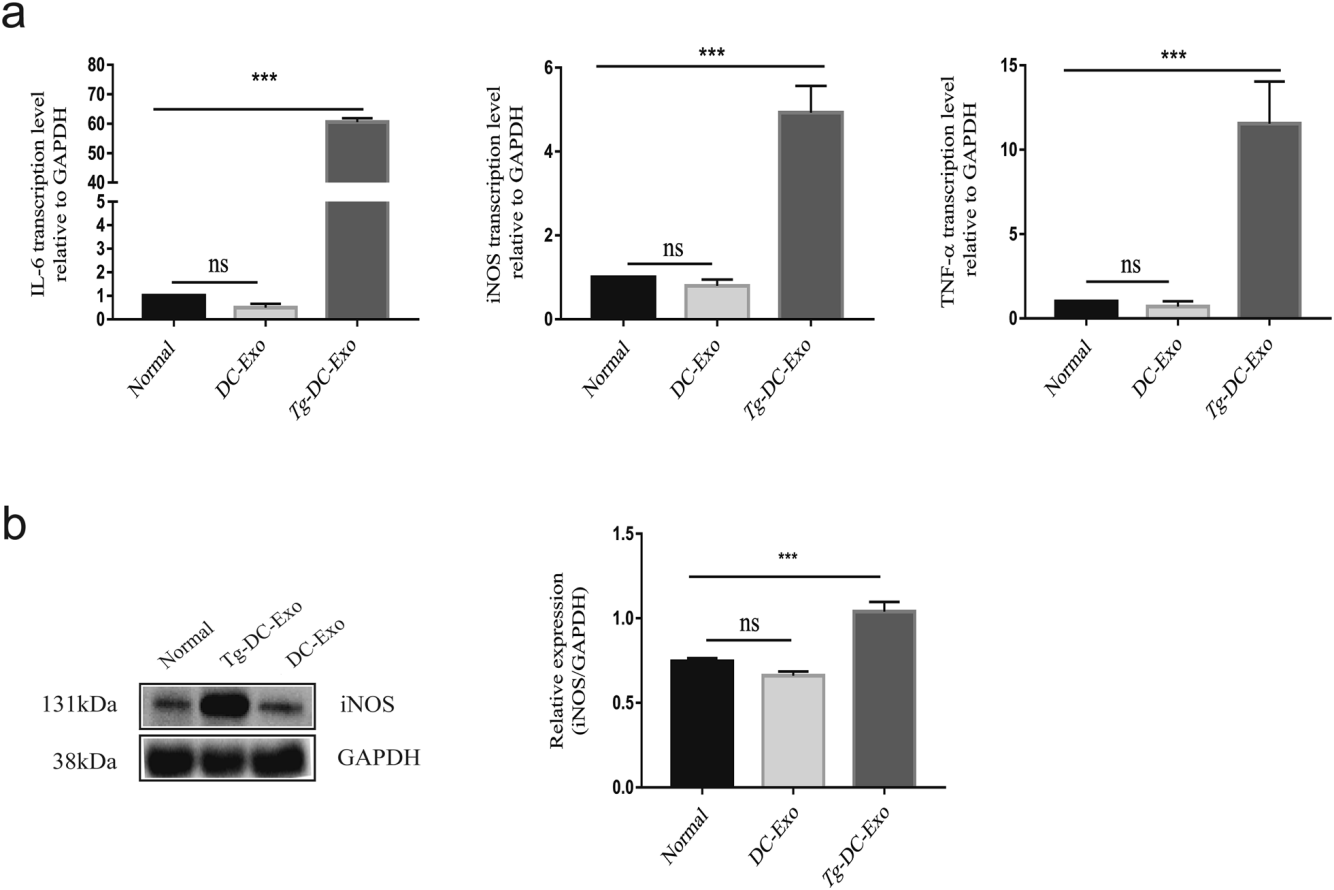
Observation of the different macrophage polarization stimulated by *Tg-*DC-Exo and DC-Exo. **a** The relative transcription levels of IL-6, iNOS, and TNF-α in the RAW264.7 cells were quantified with qRT-PCR after treatment with 120 µg/ml *Tg-*DC-Exo or DC-Exo for 24 h, which are represented as the fold change relative to the glyceraldehyde-3-phosphate dehydrogenase (GAPDH) level (set to 1). **b** The proinflammatory marker (iNOS) was detected by western blot. Each band densitometric quantitation in **b** was applied using ImageJ software. Each experiment was carried out three times for statistical analysis. The differences between two or three groups were analyzed with GraphPad Prism (GraphPad Software) or SPSS (version 20) using one-way analysis of variance (ANOVA); Tukey’s multiple-group test was used for multiple-group comparisons (***P < 0.001)

Based on the observation that RAW264.7 cells ingested exosomes secreted by DC2.4 cells, we proposed that exosomes were involved in the intercellular communication between different types of immune cells. To test this hypothesis, we treated RAW264.7 cells with *Tg*-DC-Exo or DC-Exo for 24 h, and then investigated the transcription levels of the proinflammatory cytokines (TNF-α, IL-6) and proinflammatory marker iNOS relative to which of the housekeeping gene-glyceraldehyde-3-phosphate dehydrogenase (GAPDH) were detected with real-time PCR. The results showed that the transcription levels of IL-6 (*F*_(2,6)_ = 4043, *P *< 0.0001), iNOS (*F*_(2,6)_ = 113.5, *P * < 0.0001), and TNF-α (*F*_(2,6)_ = 53.91, *P* < 0.0001) were increased in the RAW264.7 cells treated with *Tg*-DC-Exo compared with those of the cells treated with DC-Exo (Fig. 3a). Consistent with the RT-PCR results, in our WB detection, *Tg*-DC-Exo promoted iNOS translation compared with DC-Exo treatment and nontreatment (Fig. 3b)(*F*_(2,6)_ = 83.47, *P * < 0.0001). Taken together, these results indicated that *Tg*-DC-Exo could induce RAW264.7 M1 macrophage polarization in vitro.

### MiR-155-5p is abundantly packed in *Tg*-DC-exo and delivered to recipient cells via exosomes

**Fig. 4:**
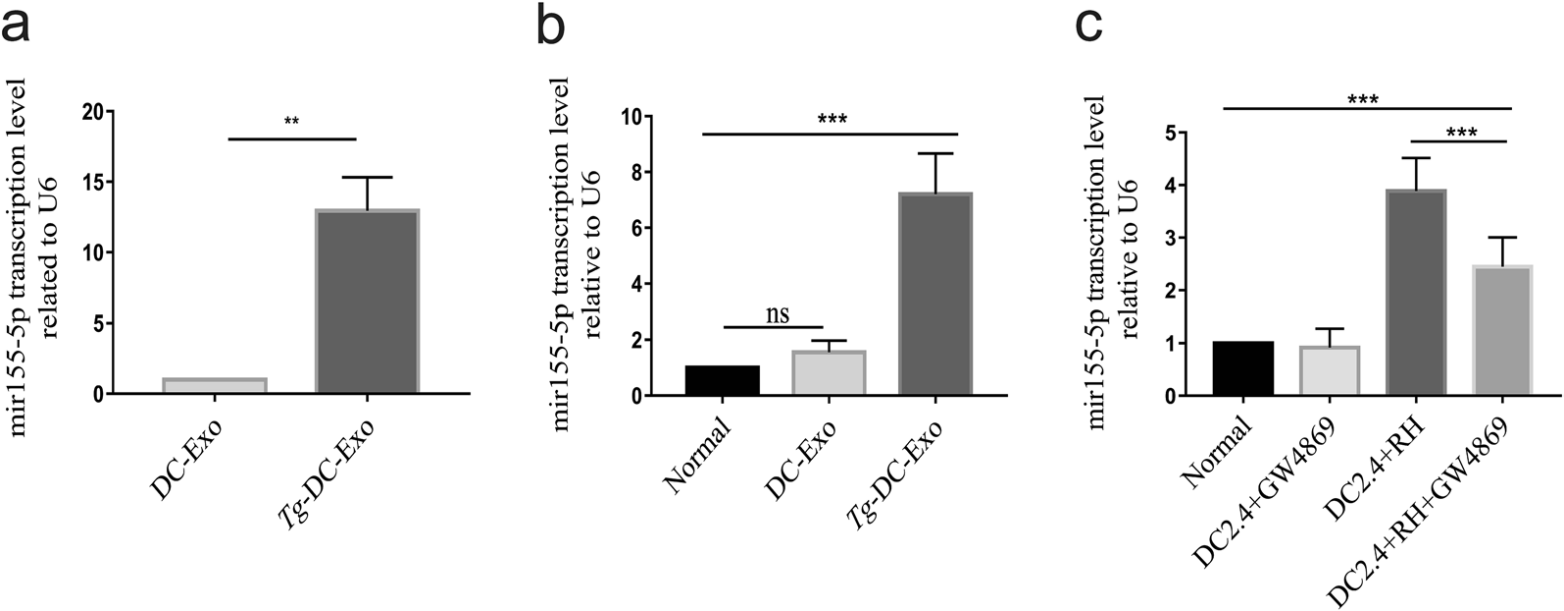
Evidence of miR-155-5p being enriched in *Tg*-DC-Exo and delivered to recipient cells via exosomes. **a** qRT-PCR confirmation of the abundance of miR-155-5p in *Tg*-DC-Exo and DC-Exo. **b** After RAW264.7 cells were treated with 120 µg/ml *Tg*-DC-Exo or DC-Exo for 24 h, the miR-155-5p transcription levels were quantified with qRT-PCR and represented as the fold change relative to the U6 level (set to 1); normal cells were set as the negative control. **c** DC2.4 cells were pre-cultured in upper transwell chambers overnight. As indicated, the DC2.4 cells were pretreated with GW4869 or not treated, followed by infection with RH tachyzoites for 30 min or non-infection, and then the unrecruited tachyzoites were washed off. Synchronously, RAW264.7 cells were cultured in the lower chambers together with the infected DC2.4 cells in the upper chambers, in the same well for 28 h. The miR-155-5p transcription levels in each lower chamber were detected with qRT-PCR, and represented as the fold change relative to the U6 level (set to 1); normal cells were set as the negative control. Data are presented as mean ± standard deviation (SD). Student’s *t*-test and one-way ANOVA were used for the significance analysis, and Tukey’s multiple-group test was used for multiple-group comparisons. Each experiment was carried out three times for statistical analysis. (** *P* < 0.01 and ***P < 0.001)

To examine the potential regulatory roles of dendritic cell exosomes taken up by recipient cells, we identified 19 differentially enriched exosomal miRNAs from the DC2.4 cells infected with *T. gondii* RH tachyzoites using RNA-seq [27]. To gain further insight into the potential regulatory roles of *Tg*-DC-Exo miRNA cargo in recipient cells, we selected the abundant miR-155-5p for further analysis. We first confirmed the transcriptome sequencing result of the upregulated miR-155-5p expression in the *Tg*-DC-Exo group compared with that in the DC-Exo group with qRT-PCR (Fig. 4a)(*F* = _(2,6)_ 777 _(2,6)_ = 113.5, *P* < 0.0001) = t_(3)_ = 6.733, *P* = 0.0067). To determine whether miR-155-5p could be transported to the recipient cells through the exosomes secreted by the DC 2.4 cells infected with *T. gondii*, RAW264.7 cells were cocultured with *Tg*-DC-Exo or DC-Exo for 24 h, and the abundance of miR-155-5p was detected with qRT-PCR. The level of miR-155-5p relative to U6 increased by several times in *Tg*-DC-Exo-treated RAW264.7 cells compared with that in DC-Exo-treated RAW264.7 cells (Fig. 4b) (*F*_(2,6)_ = 46.06, *P* = 0.0002).

In our transwell experiment, DC2.4 cells infected with *T. gondii* and uninfected DC2.4 cells were pretreated with GW4869 (an inhibitor of exosome secretion) for 24 h to prevent the production of exosomes, or were left untreated with GW4869 as a control. Next, RAW264.7 cells were cultured in the lower chambers for 28 h and then harvested for detection of miR-155-5p transcription with qRT-PCR. We found that the relative transcription level of miR-155-5p in the RAW264.7 cells was significantly reduced in the DC2.4 + RH + GW4869 treatment group compared with that in the DC + RH treatment group (Fig. 4c)(*F*_(3,16)_ = 47.01, *P* < 0.0001). All these data indicated that the miR-155-5p enriched in the exosomes derived from DC2.4 cells infected with *T. gondii w*as transported to and absorbed in the RAW264.7 cells.

### The miR-155-5p packed in the exosomes secreted by DC2.4 cells infected with *T. gondii* (*Tg*-DC-exo) modulates macrophage polarization

**Fig. 5:**
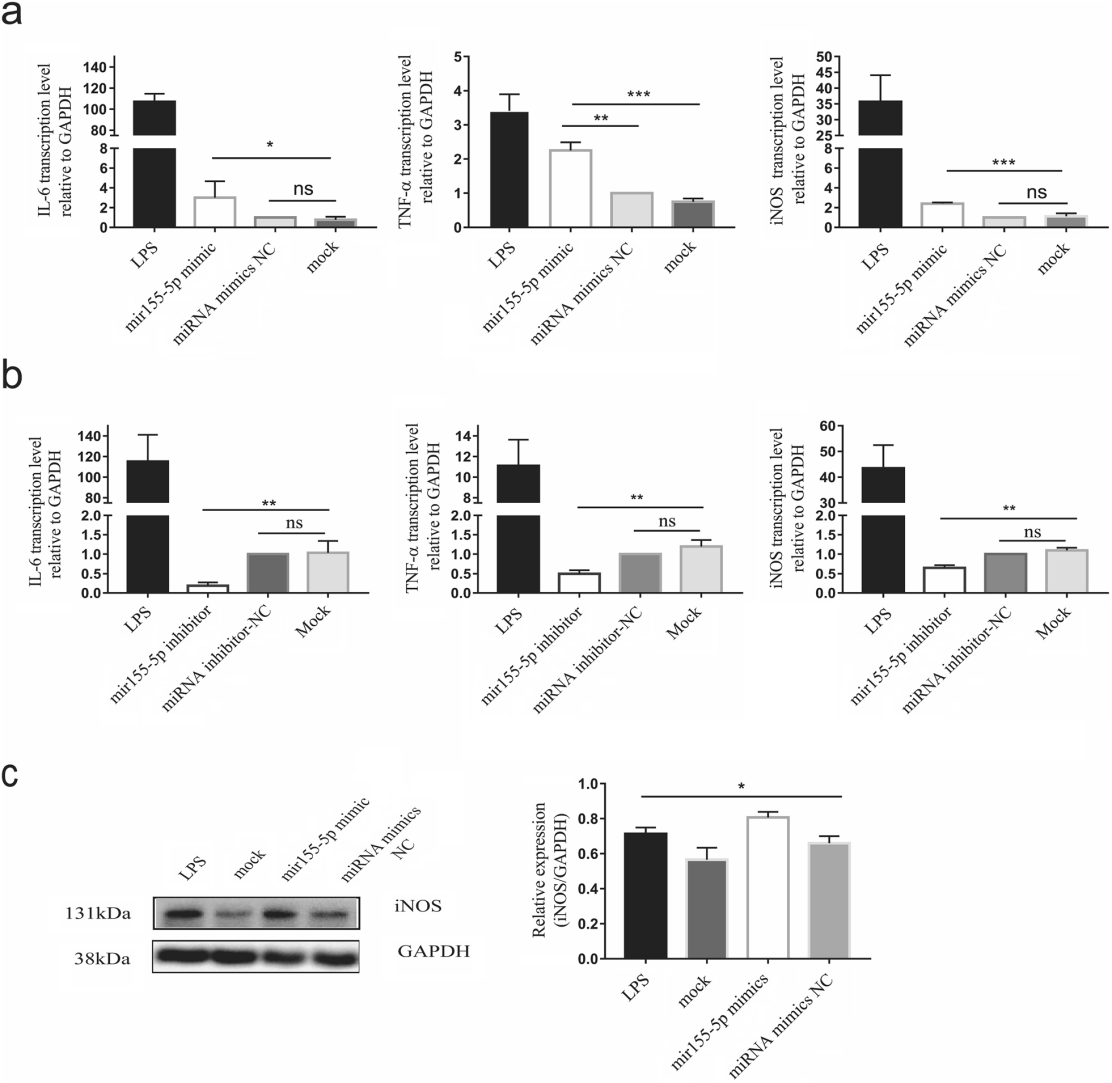
*Tg*-DC-Exo-enriched miR-155-5p promotes macrophage M1 polarization. After the indicated treatment, the levels of mRNAs in RAW264.7 cells were quantified with qRT-PCR and are represented as the fold change relative to U6 (set to 1) for miR-155-5p or relative to GAPDH (set to 1) for IL-6, iNOS, and TNF-α. The relative transcription levels of IL-6, iNOS, and TNF-α in the miR-155-5p mimic-transfected RAW264.7 cells at 18 h post-transfection (**a**) and in the miR-155-5p inhibitor-transfected cells at 24 h post-transfection (**b**). Western blot assay for iNOS expression in the miR-155-5p mimic transfection group and the other groups as control at 48 h post-transfection (**c**, left). Densitometric quantitation of each band in **c**-left was applied using ImageJ software (**c**, right). The mimics control transfection, and mock cells were set as negative controls, and the lipopolysaccharide (LPS) treatment was set as the positive control. SPSS software was used for statistical analysis. One-way ANOVA was used for between-group comparisons, and Tukey’s multiple-group test was used for multiple-group comparisons. Each experiment was carried out three times (*P < 0.05, **P < 0.01 and ***P < 0.001)

In this study, we found that miR-155-5p was highly enriched in *Tg*-DC-Exo (*t*_(3)_ = 6.733, *P* = 0.0067) and *Tg*-DC-Exo-induced RAW264.7 M1 polarization (*P* < 0.0001). We therefore hypothesized that miR-155-5p could be a key factor that plays an important role in *Tg*-DC-Exo-induced M1 polarization of RAW264.7. We first transfected miR-155-5p mimics and miR-155-5p inhibitors into RAW264.7 cells and miR-155-5p inhibitors into DC2.4 cells, and consistent levels of miR-155-5p were detected in the transfected RAW264.7 cells with qRT-PCR (Additional file 2: Figure S1a)(*P* = 0.011). Proliferation of these RAW264.7 cells was detected with a CCK8 kit at different times post-transfection. However, no significant differences were found in cell proliferation and viability at different time points among these transfected or un-transfected RAW264.7 cells (Additional file 2: Figure S1b)(*F*_(2,6)_ = 28.4, *P* = 0.0001). We further speculated that miR-155-5p modulated the polarization direction of macrophages. We found in our experiments that miR-155-5p mimics led to significant upregulated transcription levels of proinflammatory cytokines IL-6 (*F*_(3,8)_ = 533.4, *P* = 0.0246) and TNF-α (*F*_(3,8_) = 61.5, *P* = 0.0022) and proinflammatory marker (iNOS)(*F*_(3,8)_ = 49.2, *P* = 0.0002) in RAW264.7 cells compared to that in miRNA mimic-NC and mock treatment cells (Fig. 5a). To further verify the role of miR-155-5p in macrophages, we transfected a miR-155-5p inhibitor to DC2.4 and then extracted the exosomes (miR-155-5p inhibitor-DC-Exo). The miR-155-5p inhibitor-DC-Exo and the exosomes extracted from the normal DC2.4 cells (negative control-Exo) were added to RAW264.7 cells and incubated for 24 h, after which the cytokines in the culture medium were detected. However, no significant differences in cytokine levels were found between these groups (Additional file 3: Figure S2). Considering the low background level of miR-155-5p [32] and loss of exosomes in extraction, the inhibition effect of the miR-155-5p in exosomes may be unobservable. Therefore, we further transfected a miR-155-5p inhibitor to RAW264.7 cells and found it significantly inhibited the transcription of IL-6, iNOS, and TNF-α in RAW264.7 cells compared to that in miRNA-NC inhibitor (miRNA normal control inhibitor) treatment and mock treatment groups (Fig. 5b)(*P* = 0.001). WB showed a result consistent with that of the qRT-PCR: miR-155-5p mimics significantly upregulated iNOS expression in RAW264.7 cells compared to miRNA mimic-NC and mock treatments (Fig. 5c)(*F*_(3,8)_ = 14.14, *P* = 0.0015). All of the evidence illustrated that *Tg*-DC-Exo-derived miR-155-5p contributed to M1 macrophage polarization.

### The miR-155-5p packed in *Tg*-DC-exo inhibits the multiplication of *T. gondii* RH tachyzoites

**Fig. 6:**
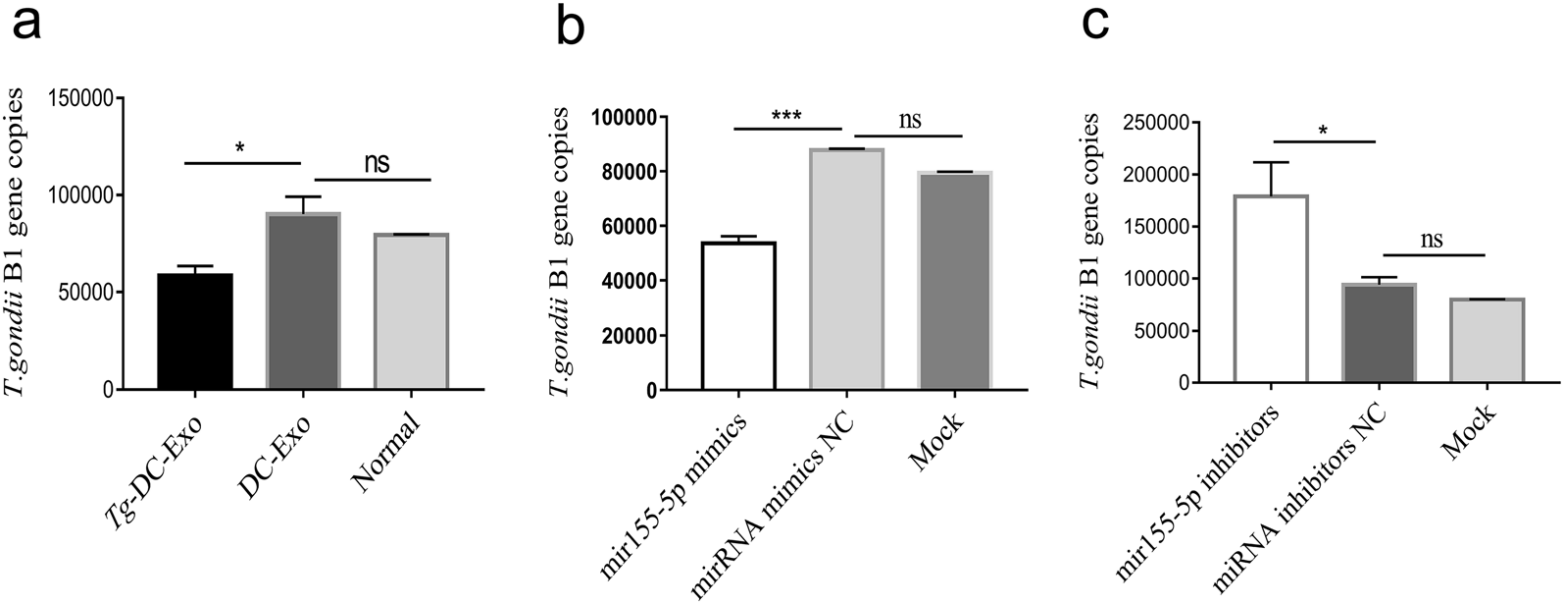
Inhibited proliferation of *T. gondii* in the *Tg*-DC-Exo treatment and miR-155-5p transfection groups. The *T. gondii* B1 gene copies were quantified with qPCR. **a** The RAW264.7 cells were treated with 120 µg/ml *Tg*-DC-Exo or DC-Exo for 24 h and then infected with *T. gondii* for 24 h. The RAW264.7 cells were transfected with miR-155-5p mimics (**b**) or miR-155-5p inhibitors (**c**) for 24 h and then infected with *T. gondii* for 24 h; the mimic control transfection and mock cells were set as negative controls. SPSS software was used for statistical analysis. One-way ANOVA was used for between-group comparisons, and Tukey’s multiple-group test was used for multiple-group comparisons. Each experiment was carried out three times (*P < 0.05, ***P  < 0.001)

Since increased production of proinflammatory factors inhibited *T. gondii* multiplication, and since we had identified that *Tg*-DC-Exo-derived miR-155-5p upregulated the transcription of some proinflammatory cytokines (Figs. 3 and 5), we wondered whether the *Tg*-DC-Exo-derived miR-155-5p inhibited *T. gondii* multiplication or not. We found that *Tg*-DC-Exo treatment on RAW264.7 cells resulted in the inhibition of *T. gondii* replication in the RAW264.7 cells compared to that in DC-Exo treatment and nontreatment groups (Fig. 6a)(*F*_(2,6)_ = 14.74, *P* = 0.028). We therefore assumed that the *Tg*-DC-Exo significantly enriched with miR-155-5p also had the same effect on *T. gondii* replication. To test this hypothesis, firstly, a CCK8 kit was used to examine the cell activity affected by miR-155-5p mimics or miR-155-5p inhibitors, and no significant differences were found between the RAW264.7 cell groups which were transfected or un-transfected with these miRNAs (Additional file 4: Figure S3). Secondly, the *T. gondii* B1 gene copies (indicating the number of *T. gondii* tachyzoites) in the cells transfected with miR-155-5p mimics or miR-155-5p inhibitors were evaluated with qRT-PCR. The results showed us that the B1 gene copies were significantly lower in the RAW264.7 cells transfected with the miR-155-5p mimic than in the cells transfected with the miRNA mimic-NC and in the mock cells (Fig. 6b)(*F*_(2,6)_ = 620.8, *P* = 0.0004). On the contrary, the B1 gene copies were significantly higher in the RAW264.7 cells transfected with miR-155-5p inhibitors than in the cells transfected with the miRNA-NC inhibitor and the mock cells (Fig. 6c)(*F*_(2,6)_ = 15.22, *P* = 0.009). In brief, these results indicated that *Tg*-DC-Exo treatment and the high level of miR-155-5p in RAW264.7 cells significantly inhibited the multiplication of *T. gondii* in these cells.

### *Tg*-DC-exo-derived miR-155-5p targets *socs1* to activate the NF-κB signaling pathway in macrophages

**Fig. 7:**
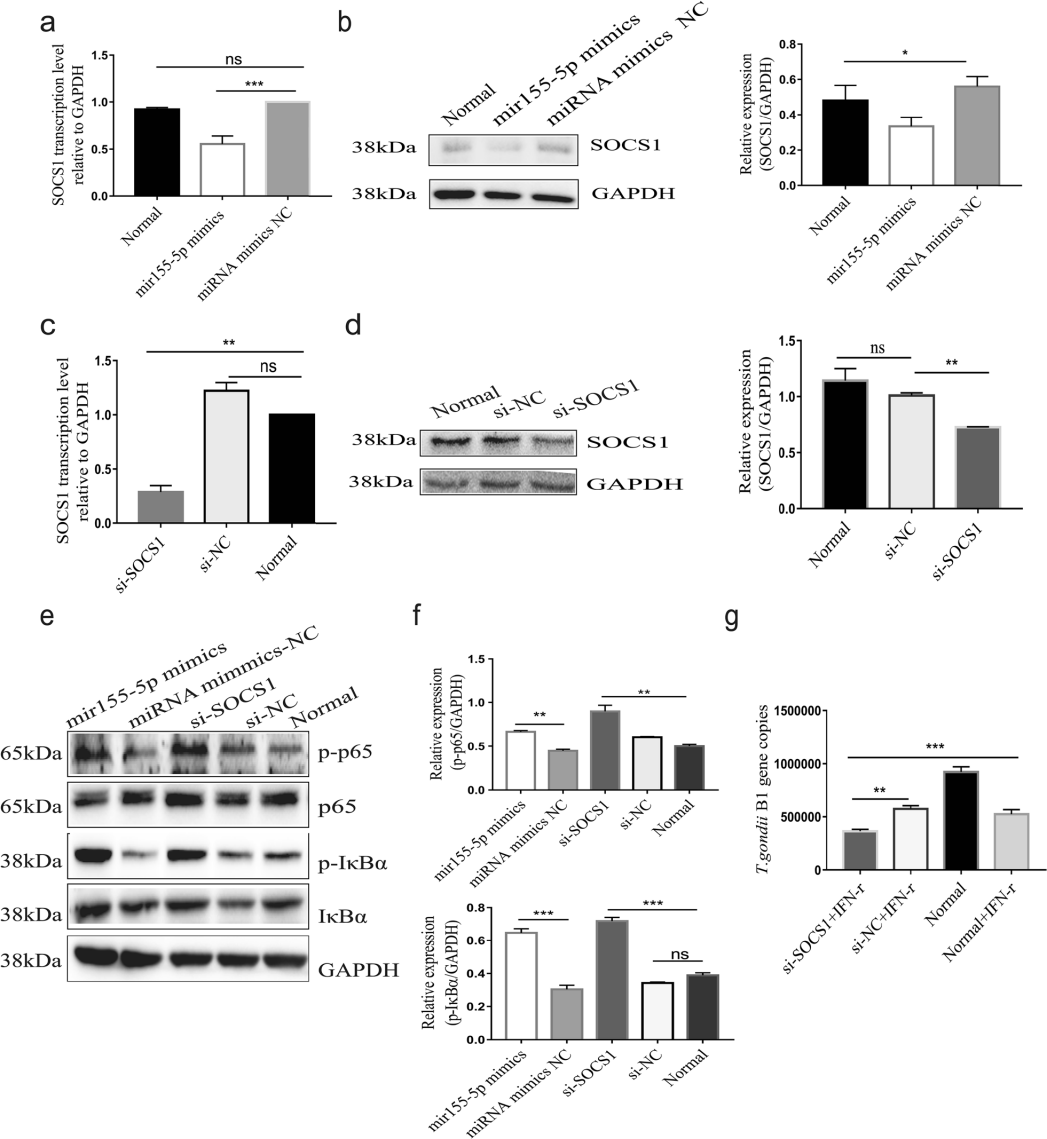
*Tg*-DC-Exo miR-155-5p targeting *socs1* to promote the inhibitory effect of interferon-γ (IFN-γ) on *T. gondii* proliferation through the NF-κB pathway. Detection of *socs1* transcription with qRT-PCR at 24 h post-transfection and translation with western blot at 48 h post-transfection in RAW264.7 cells. **a**, **b** Transfection with miR-155-5p mimics or normal control miRNA (mimic-NC). **c**, **d** Transfection with the siRNA targeting *socs1* (si-*socs1*) and the normal control siRNA (si-NC). **e**, **f** Detection of the phosphorylation levels of p65 and IKBα in the NF-κB pathway in the RAW264.7 cells transfected with miR-155-5p mimics, normal control siRNA (mir-NC), si-*socs1*, or si-NC for 36 h. **g** Detection of the *T. gondii* B1 gene copies in the RAW264.7 cells transfected with si-*socs1* or si-NC for 24 h, and stimulated with IFN-γ for 24 h, then infected with *T. gondii* for 24 h. Densitometric quantitation of each band in **b**, **d**, and **e** was applied using ImageJ software. One-way ANOVA was used for between-group comparisons, and Tukey’s multiple-group test was used for multiple-group comparisons. Each experiment was carried out three times (**P < 0.01, ***P < 0.001)

To identify the target genes of miR-155-5p in RAW264.7, online bioinformatics tools (TargetScan and miRWalk) were used to predict the potential target genes. Among the potential targets, the suppressor of cytokine signaling (*socs1*), which is involved in both immune modulation and cell cycle regulation, has been reported to be a negative feedback regulator of cytokines and to be involved in multiple signaling pathways [33]. Hence, we assumed that *socs1* was targeted by miR-155-5p and acted to promote macrophage M1 polarization. We found that the transcription and translation of *socs1* were significantly inhibited by miR-155-5p mimics compared to that in miRNA mimic-NC transfection and non-transfection groups (Fig. 7a(*F*_(2,6)_ = 54.77, *P* = 0.0004), b(*F*_(2,6)_ = 9.041, *P* = 0.0136). Considering the fact that the activation of NF-κB plays a crucial role in M1 macrophage polarization and inhibition of *T. gondii* proliferation [34], we speculated that the miR-155-5p packed in *Tg*-DC-Exo was transported to RAW264.7 cells and then targeted *socs1* to activate the NF-κB signaling pathway. To test our hypothesis, *socs1* was knocked down in RAW264.7 cells using small interfering RNAs targeting *socs1* (si-*socs1*) (Fig. 7c(*F*_(2,6)_ = 153.2, *P* = 0.001), d(*F*_(2,6)_ = 31.26, *P* = 0.005). We further found that miR-155-5p mimics and si-*socs1* transfection promoted the phosphorylation of IKBα and p65 in RAW264.7 cells compared with the si-NC transfection and un-transfected groups (Fig. 7e, f)(*P* < 0.0001). IKBα and p65 phosphorylation represent the activation of NF-κB signaling; as a result, macrophage M1 polarization was subsequently induced. We also found that si-*socs1* transfection to RAW264.7 cells did not affect the cell proliferation or viability (Additional file 5: Figure S4). Next, we investigated the role of SOCS1 in *T. gondii* RH proliferation in RAW264.7 cells by detecting *T. gondii* B1 gene copies in si-*socs1* or si-NC transfected groups with qRT-PCR. No significant differences in *T. gondii* RH proliferation were found between the groups (Additional file 5: Figure S4c). Furthermore, when pretreated with IFN-γ, si-*socs1* played a synergistic role in the inhibition of *T. gondii* proliferation (Fig. 7g)(*F*_(3,8)_ = 105.2, *P* < 0.0001). Collectively, these results demonstrated that miR-155-5p targeted the *socs1* gene to activate the NF-κB signaling pathway and inhibited the proliferation of *T. gondii* in RAW264.7 cells.

## Discussion

Host immune sensors recognize *T. gondii* infection and activate immune cells to produce proinflammatory cytokines against infection [35–37]. Here, we found that exosomes derived from dendritic cells could be ingested by RAW264.7 cells, and miR-155-5p was highly enriched in exosomes derived from dendritic cells infected with *T. gondii* (*Tg*-DC-Exo), but not in the exosomes from normal dendritic cells (DC-Exo). MiR-155-5p was found to target the *socs1* gene to activate the NF-κB pathway, and subsequently inhibited *T. gondii* proliferation in RAW264.7 cells.

Exosomes, which are released by most cell types and pathogens and are involved in cell-to-cell communication, have been extensively studied in parasitic infection and disease progression [38]. It has been shown that abnormal cells produce more extracellular vesicles (EVs) than normal cells [39, 40]. In our study, the exosomes derived from *T. gondii*-infected DC2.4 cells (*Tg*-DC-Exo) were found with higher particle concentration than those derived from normal DC2.4 cells (DC-Exo) (Fig. 1d), which is similar to the report by Wowk et al. [13].

During the phase of acute infection, the anti-*Toxoplasma* effect is mainly mediated by macrophage activation by IFN-γ and cytotoxicity mediated by CD8^+^ T cells, which are attributed to the production of IFN-γ and other inflammatory factors [41–43]. On the other hand, perforin was found to mediate cytotoxic activity of CD8^+^ T cells in the clearance of *T. gondii* cysts during chronic infection [44]. The RAW264.7 cell line has been used as a model for observing the intercellular transmission of infection signals between different types of immune cells in many reports [45–47]. In our study, we found that the intercellular communication between dendritic cells and macrophages could be realized through exosome transmission (Fig. 2). This could be a mechanism by which infection signals from infected cells are transmitted to uninfected cells.

*Toxoplasma gondii* infection altered the miRNA profiles of exosomes secreted from DC2.4 cells; for example, miR-146a, let7k, and miR-155-5p were upregulated in DC2.4 cells after *T. gondii* infection [27]. It has been reported that miR-155 is sensitive to pathogen stimulation, abnormally expressed in activated dendritic cells, macrophages, T cells, and B cells, and induces a robust proinflammatory response in macrophages [32, 48]. In our research, we found that RAW264.7 cells absorbed exosomes secreted by DC2.4 cells, and miR-155-5p was abundantly enriched in *Tg*-DC-Exo and delivered to RAW264.7 cells via exosomes to activate the immune signaling pathways. Given the unique functions of miR-155 in the immune system, such as controlling inflammatory responses, regulating immune memory, and so on [49, 50], the regulation of immune responses by miR-155-5p in RAW 264.7 cells was further investigated.

We found that *Tg*-DC-Exo and miR-155-5p mimics boosted the transcription levels of IL-6, iNOS, and TNF-α, which are macrophage M1-associated markers (Figs. 3 and 5). In *T. gondii* (RH) infection, dendritic cells transmitted immune signals to macrophages by secreting exosomes enriched with miR-155-5p. As a result, the macrophages were driven to M1 polarization and participated in the positive immune responses after receiving miR-155-5p. However, it has been reported that *T. gondii* (types I and III) direct infection drives the polarization of macrophages towards M2 [51]. It is possible that *T. gondii* (types I and III) ROP16 activates M2 macrophages by activating STAT6, which reduces the secretion of proinflammatory factors such as IL12 in the early infection stage, thus helping to reduce the host responses.

It is generally believed that M1 macrophages exert positive immunomodulatory effects and inhibit *T. gondii* multiplication by secreting proinflammatory cytokines TNF-α and IL-6 and proinflammatory marker iNOS [43, 52]. Therefore, the inhibited proliferation of *T. gondii* was verified in the RAW264.7 cells transfected with miR-155-5p mimics (Fig. 6). We previously reported that the target genes of the significantly enriched miRNAs in *Tg*-DC-Exo were mainly related to immunity and aggregated in NF-κB, MAPK, P13K–AKT, and the calcium signaling pathway [27]. It has also been reported that the immune functions of miR-155 fall mainly into the regulation of the *socs1* gene [53]. We further verified that *socs1* was the target gene of miR-155-5p through an experiment with miR-155-5p mimic transfection (Fig. 7a, b), and this result was consistent with that of the previous report by Ye et al. [54]. It has been shown that, as a negative feedback cytokine regulator, SOCS1 is involved in multiple immune-related signaling pathways [55–58]; on the other hand, as the NF-κB signaling pathway is crucial for inflammatory response regulation, it can be activated by a variety of proinflammatory factors [59]. In our studies, we found that the NF-κB signaling pathway was significantly activated after miR-155-5p mimics were transfected into RAW264.7 cells (Fig. 7e). However, the modulation of *socs1* by miR-155-5p may also affect cellular pathways other than the NF-κB pathway. Therefore, the function of the *Tg*-DC-Exo-enriched miR-155-5p in anti-*T. gondii* infection remains to be further investigated.

Host IFN-γ plays a crucial role in the control of *T. gondii* infection [60, 61]. We found in our research that SOCS1, an effective inhibitor of the IFN-γ pathway, showed an effect on the proliferation of *T. gondii* in RAW264.7 cells; when the RAW264.7 cells were treated with IFN-γ, the proliferation of *T. gondii* in the *socs1* knockdown group was significantly more inhibited than in the normal control siRNA (si-NC) transfected cells and the normal control cells (both stimulated or not stimulated by IFN-γ) (Fig. 7g). This result implied an inhibitory role of SOCS1 in IFN-γ anti-infection, which is consistent with the report that *T. gondii* induces host cell *socs1* expression to block IFN-γ signaling for immune evasion [62].

## Conclusions

The exosomes secreted by DC2.4 cells infected with *T. gondii* (*Tg*-DC-Exo) transmitted *T. gondii* infection signals to RAW264.7 cells through the latter’s exosome uptake. The enriched miR-155-5p in *Tg*-DC-Exo targeted the *socs1* gene to activate the NF-κB pathway and then to promote the transcription of inflammatory factors including TNF-α, IL-6, and iNOS. As a result, *T. gondii* proliferation was inhibited in these RAW264.7 cells. This finding may provide new insight into intercellular communication for the transmission of infection signals between host cells, and may have implications for the role of exosomal miR-155-5p in anti-*T. gondii* infection.

## Supplementary Information

**Additional file 1: Table S1.** Primers and siRNA, inhibitors, mimics sequences list.

**Additional file 2: Figure S1.** Detection of miR-155-5p level and the cell proliferation in differently treated immune cells. **a** Detection of the miR-155-5p level in the indicated cells at 24 h post-transfection. Left: RAW264.7 cells of normal, miR-155-5p mimics transfected, miRNA-mimic-NC transfected, miR-155-5p inhibitor transfected, miRNA-inhibitor-NC transfected, and mock. Right: DC2.4 cells of normal, miR-155-5p inhibitor transfected, and miRNA-inhibitor-NC transfected. **b** RAW264.7 cell proliferation was detected after transfection with miR-155-5p mimics or miR-155-5p inhibitors for 12, 24, 48, 72, and 96 h, as indicated. The mimic control, inhibitor control, and normal cells were used as negative controls. One-way ANOVA was used for between-group comparisons, and Tukey’s multiple-group test was used for multiple-group comparisons. Each experiment was carried out three times (*P < 0.05, ****P < 0.0001)

**Additional file 3: Figure S2.** Detection of IL-6, iNOS, and TNF-α transcription level in the RAW 264.7 cells treated with differently derived exosomes. The relative transcription levels of IL-6, iNOS, and TNF-α were detected in the RAW264.7 cells treated with the exosomes derived from the DC2.4 cells transfected with miR-155-5p inhibitor (miR-155-5p inhibitor-Exo) or miRNA inhibitor NC (miRNA inhibitor NC-Exo) at 24 h post-transfection.

**Additional file 4: Figure S3.** The cell viability detection of the RAW264.7 cells transfected with miR-155-5p mimics or inhibitors at 24 h post-transfection.

**Additional file 5: Figure S4.** Detection of the cell proliferation and viability of the RAW264.7 cells after different treatment. **a** RAW264.7 cell proliferation was detected after transfection with si-*socs1*, and si-NC for 12, 24, 48, 72, and 96 h. **b** The cell viability detection of the RAW264.7 cells transfected with si-*socs1*, and si-NC for 24 h. **c** Detection of the *T. gondii* B1 gene copies in the RAW267.4 cells transfected with si-*socs1* or si-NC for 24 h, then infected with *T. gondii* for 24 h.

## Data Availability

Not applicable.

## Abbreviations

*T. gondii*: *Toxoplasma gondii*
Exo: Exosomes
miRNA: MicroRNA
HFF: Human foreskin fibroblast
RAW264.7: Leukemia cells in mouse macrophage
FBS: Fetal bovine serum
*TSG101*: Tumor susceptibility gene 101
CD9: CD9 molecule
HSP70: Heat shock protein 70
CD81: CD81 molecule
LPS: Lipopolysaccharide
NF-κB: Nuclear factor kappa B
CCK8: Cell Counting Kit-8
iNOS: Inducible nitric oxide synthase
TNF-α: Tumor necrosis factor alpha
IL-6: Interleukin-6
SOCS1: Suppressor of cytokine signaling 1
