# Supplementary figures and images for: Anti-infection roles of miR-155-5p packaged in exosomes secreted by dendritic cells infected with Toxoplasma gondii
Source: Parasit Vectors. 2022 Jan 6;15:3. doi: 10.1186/s13071-021-05003-x (PMC8731220; doi:10.1186/s13071-021-05003-x)

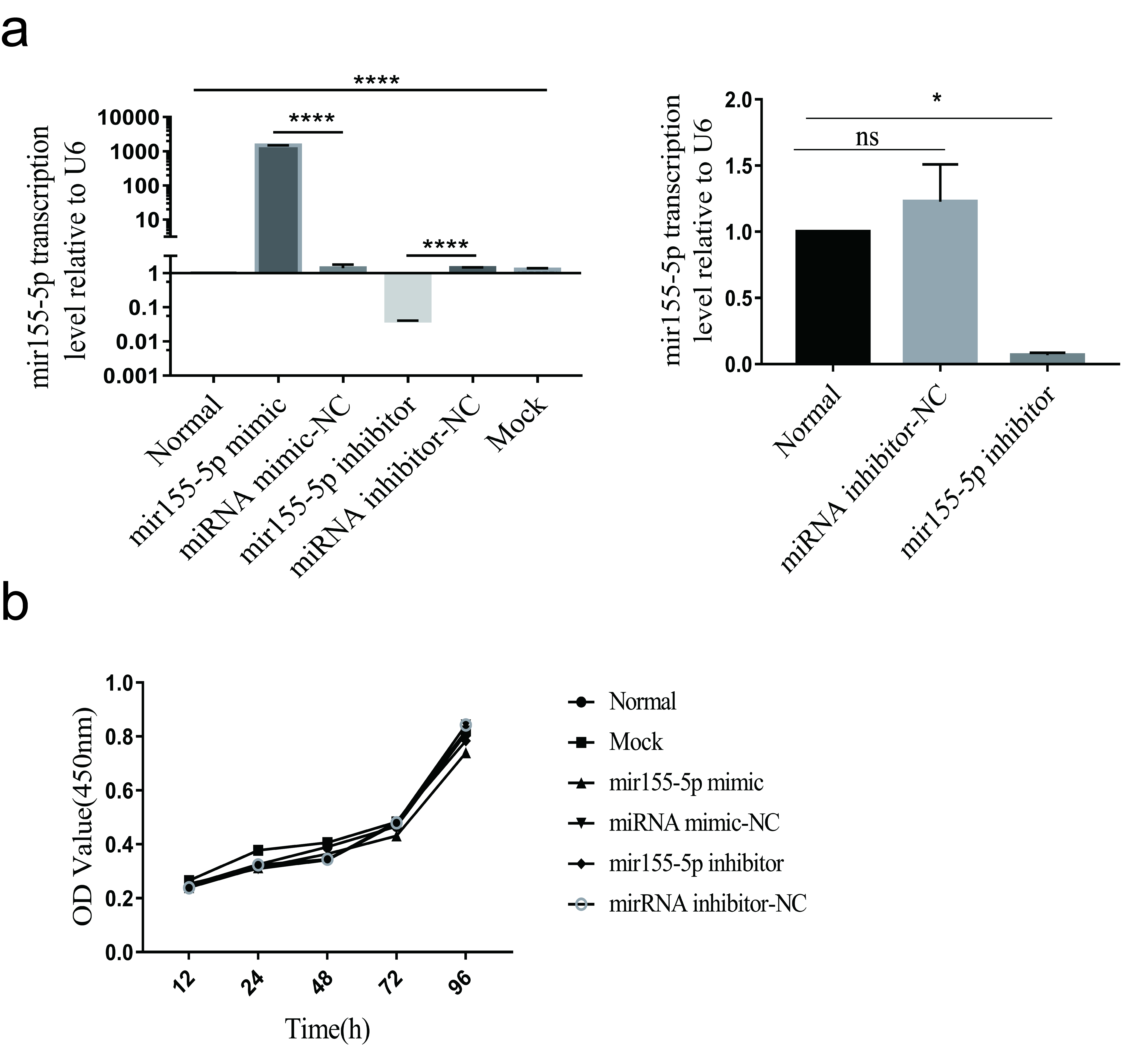

Supplement: Supplementary file 2 — Additional file 2: Figure S1. Detection of miR-155-5p level and the cell proliferation in differently treated immune cells. a Detection of the miR-155-5p level in the indicated cells at 24 h post-transfection. Left: RAW264.7 cells of normal, miR-155-5p mimics transfected, miRNA-mimic-NC transfected, miR-155-5p inhibitor transfected, miRNA-inhibitor-NC transfected, and mock. Right: DC2.4 cells of normal, miR-155-5p inhibitor transfected, and miRNA-inhibitor-NC transfected. b RAW264.7 cell proliferation was detected after transfection with miR-155-5p mimics or miR-155-5p inhibitors for 12, 24, 48, 72, and 96 h, as indicated. The mimic control, inhibitor control, and normal cells were used as negative controls. One-way ANOVA was used for between-group comparisons, and Tukey’s multiple-group test was used for multiple-group comparisons. Each experiment was carried out three times (*P < 0.05, ****P < 0.0001) [file 13071_2021_5003_MOESM2_ESM.tif]

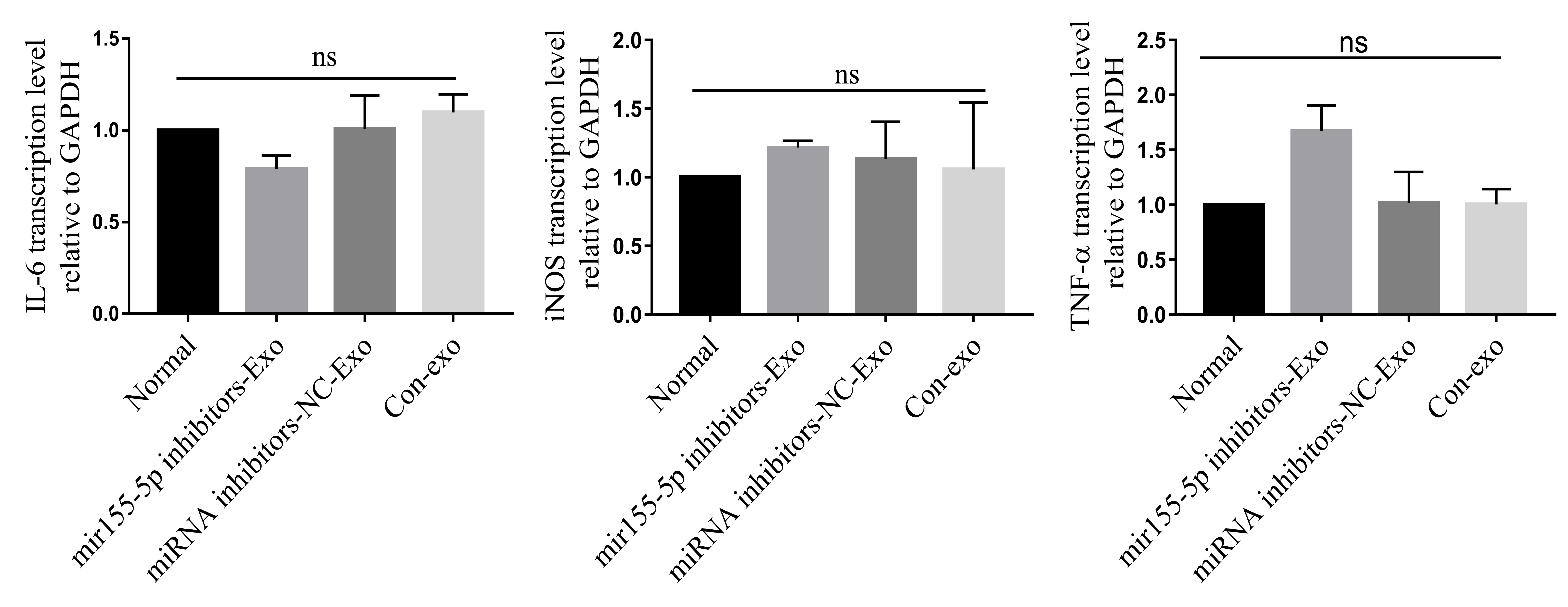

Supplement: Supplementary file 3 — Additional file 3: Figure S2. Detection of IL-6, iNOS, and TNF-α transcription level in the RAW 264.7 cells treated with differently derived exosomes. The relative transcription levels of IL-6, iNOS, and TNF-α were detected in the RAW264.7 cells treated with the exosomes derived from the DC2.4 cells transfected with miR-155-5p inhibitor (miR-155-5p inhibitor-Exo) or miRNA inhibitor NC (miRNA inhibitor NC-Exo) at 24 h post-transfection. [file 13071_2021_5003_MOESM3_ESM.tif]

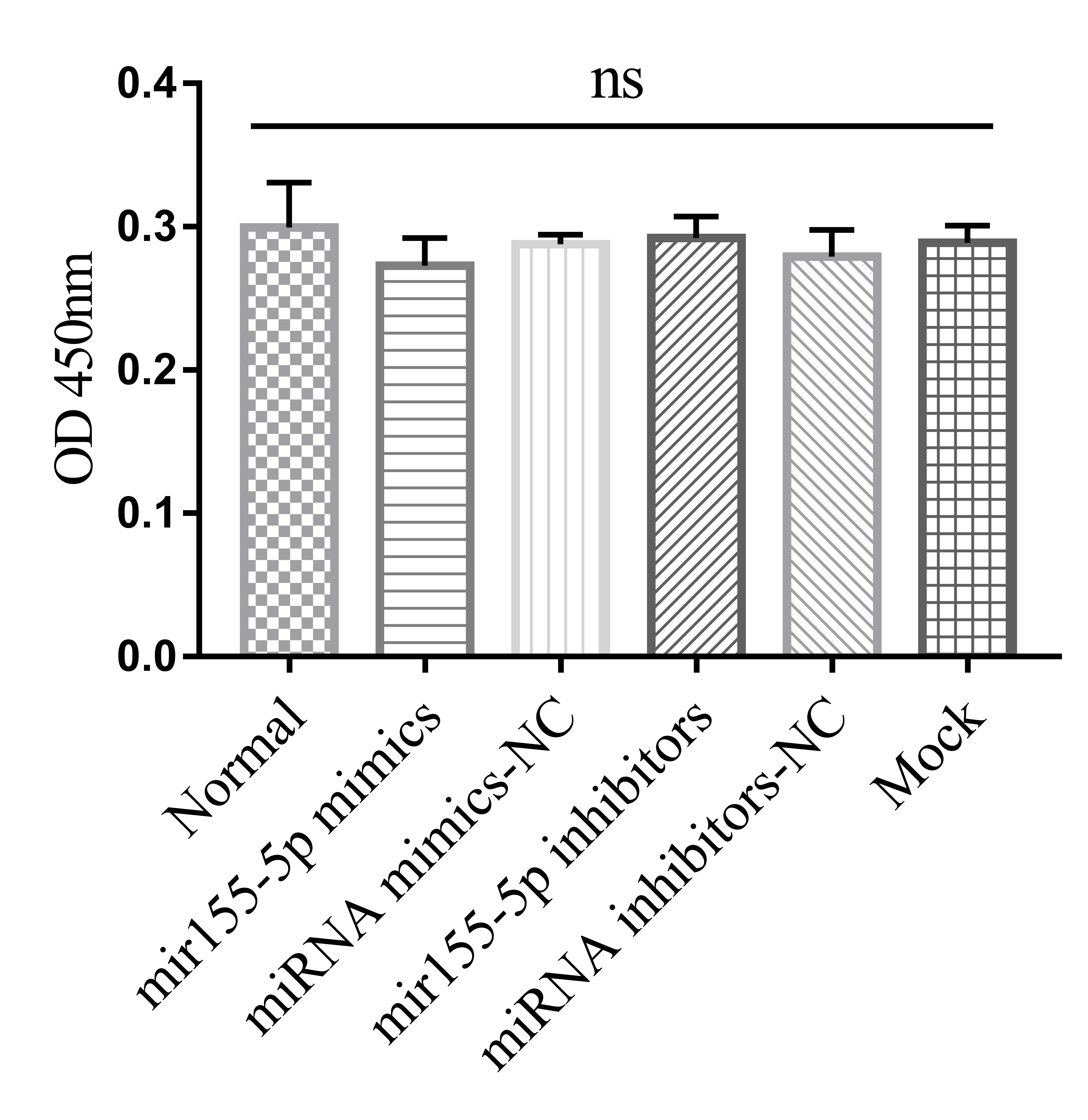

Supplement: Supplementary file 4 — Additional file 4: Figure S3. The cell viability detection of the RAW264.7 cells transfected with miR-155-5p mimics or inhibitors at 24 h post-transfection. [file 13071_2021_5003_MOESM4_ESM.tif]

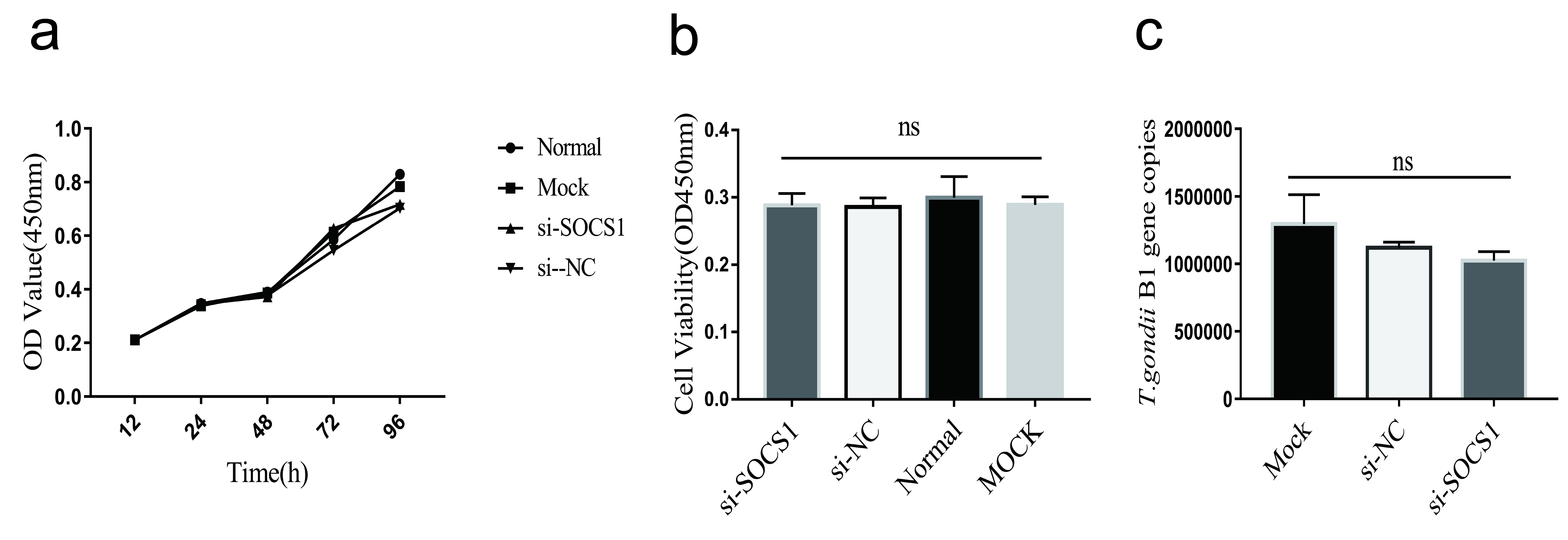

Supplement: Supplementary file 5 — Additional file 5: Figure S4. Detection of the cell proliferation and viability of the RAW264.7 cells after different treatment. a RAW264.7 cell proliferation was detected after transfection with si-socs1, and si-NC for 12, 24, 48, 72, and 96 h. b The cell viability detection of the RAW264.7 cells transfected with si-socs1, and si-NC for 24 h. c Detection of the T. gondii B1 gene copies in the RAW267.4 cells transfected with si-socs1 or si-NC for 24 h, then infected with T. gondii for 24 h. [file 13071_2021_5003_MOESM5_ESM.tif]
